# Postnatal calpain inhibition elicits cerebellar cell death and motor dysfunction

**DOI:** 10.18632/oncotarget.21324

**Published:** 2017-09-27

**Authors:** Junyao Li, Sanjuan Yang, Guoqi Zhu

**Affiliations:** ^1^ Key Laboratory of Xin’an Medicine, Ministry of Education, Anhui University of Chinese Medicine, Hefei, 230038, China

**Keywords:** postnatal, calpain inhibition, cerebellum, apoptosis, motor dysfunction

## Abstract

Calpain-1 deletion elicits neurodevelopmental disorders, such as ataxia. However, the function of calpain in postnatal neurodevelopment and its mechanisms remain unknown. In this study, we revealed that postnatal intraperitoneal injection of various calpain inhibitors attenuated cerebellar cytosolic calpain activity. Moreover, postnatal application of calpeptin (2 mg/kg) apparently reduced spectrin breakdown, promoted suprachiasmatic nucleus circadian oscillatory protein (SCOP) accumulation in cerebellar tissue. In addition, application of calpeptin decreased phosphorylated protein kinase B (p-AKT) level (p<0.05), as well as total AKT level (p<0.05). We also evidenced that administration of calpeptin obviously increased phosphorylation of mammalian target of rapamycin (p-mTor) (p<0.01). Apoptosis of granular cells and activation of caspase-3 (p<0.01) were facilitated after calpain inhibition. Importantly, cell numbers of granular cells were reduced and motor function was remarkably impaired in 4-month-old rats receiving postnatal calpain inhibition. Taken together, our data implicated that calpain activity in the postnatal period was critical for the cerebellar development. Postnatal calpain inhibition causes cerebellar granular cell apoptosis and motor dysfunction, likely through SCOP/AKT and p-mTor signaling pathways.

## INTRODUCTION

Cerebellum is an important region in the nervous system responsible for motor output and balance [1]. Abnormal development of cerebellum leads to ataxia and motor dysfunction [2]. Genetic reasons for the abnormal development of cerebellum have been well investigated. The typical examples include mutation or deletion of TSEN54, Kv3, rBAT-BAT1, UBA5 genes, etc, which subsequently cause cerebellar atrophy or dysfunction of motor ability [3–5]. Besides the genetic factors, environmental toxins, such as chronic exposure to 137cesium and nicotine also impair the prenatal or postnatal neurodevelopment [6–8]. The second week after birth is the critical period for the postnatal development [9]. In that period, the neurons are sensitive to environmental factors or endogenous cytokines [10, 11]. Although the sensitive period for postnatal neurodevelopment has been identified, the related mechanisms are still unknown.

Calpain is a type of calcium-dependent proteases, which have multiple functions in signaling transduction, synaptic plasticity and neurobehavioral activity [12–15]. However, calcium overloading activates calpain and causes irreversible neuronal death and neurodegenerative diseases [16, 17]. Therefore, calpain inhibitors have been temptingly designed to treat neurodegenerative diseases, including Parkinson's disease, Alzheimer's disease, and ischemia stroke [18–21]. Recently, calpain-mediated calcium signaling pathways have been accumulatively reported to be neuroprotective [22–24], and have also been implicated in cerebellar development [25, 26].

As a potential treatment target of neurodegenerative diseases in adults, calcium overloading was also abnormally facilitated in pathological conditions in younger animals [27, 28]. Therefore, it is still a question regarding the application of calpain inhibitors in young populations. In this study, we are interested whether calpain is required for postnatal neurodevelopment. To this end, pharmacological experiments were designed to verify the functions of calpain in the critical postnatal period. This study would provide important implications regarding the application of calpain inhibitors in the treatment of neurodegenerative diseases in children.

## RESULTS

### Postnatal application of calpain inhibitors reduces cerebellar calpain activity

**Figure 1:**
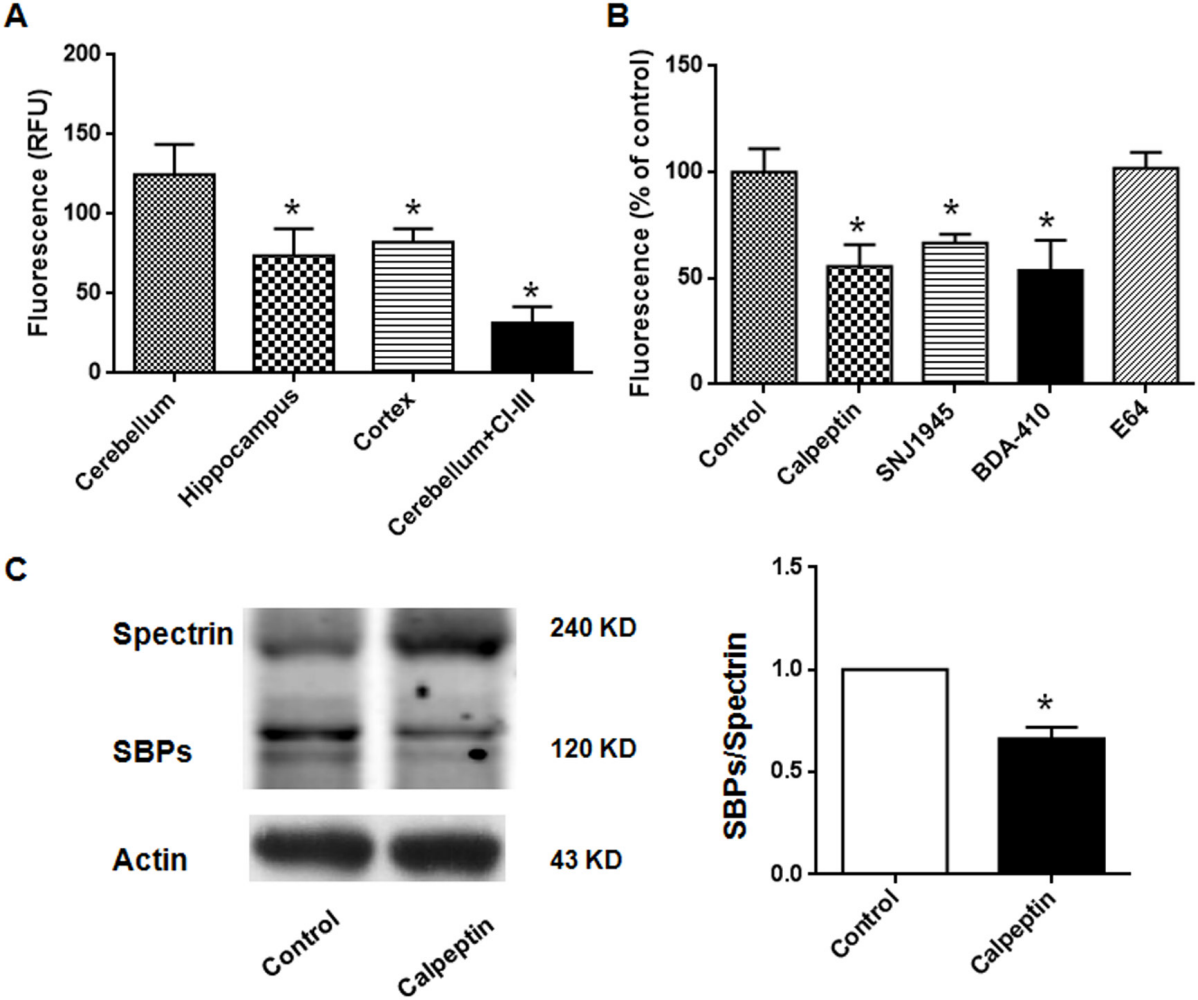
Postnatal application of calpain inhibitors reduces cerebellar calpain activity in rats **(A)** Calpain activities in different brain regions. ^*^p < 0.05 compared with cerebellum (One-way ANOVA followed by Bonferroni test). Calpain inhibitor III (1 μM) was applied in the *in vitro* experiments (Unpaired *t* test). **(B)** Calpain activities in cerebellum after *i.p.* injection of different calpain inhibitors. ^*^p < 0.05 compared with control group (One-way ANOVA followed by Bonferroni test). The dose of different calpain inhibitors were listed as following: Calpeptin (2 mg/kg), SNJ1945 (1 mg/kg), BDA-410 (1 mg/kg), E64 (5 mg/kg). **(C)** Calpeptin application decreased spectrin breakdown in cerebellum. ^*^p < 0.05 compared with control group (Paired t test).

Initially, we detected cytosolic calpain activity in different brain regions obtained from P8 rats. As shown in Figure 1A, calpain activity was significantly different in cerebellum, cortex and hippocampus (F (2, 15) = 4.165). Post-hoc analysis showed that calpain activity was lower in hippocampus and cortex, compared with cerebellum (p<0.05). The values in cerebellum, hippocampus and cortex were 124 ± 19.0 relative fluorescence units (RFU), 74.0 ± 8.5 RFU and 82.3 ± 8.5 RFU, respectively. We confirmed the specific detection of calpain activity by *in vitro* application of calpain inhibitor III. As shown in Figure 1A, calpain activity in cerebellum was significantly reduced by calpain inhibitor III (31.5 ± 5.1 RFU) (Unpaired *t* test, p < 0.05) (t = 4.75, df = 14.0). Different calpain inhibitors (calpeptin, SNJ1945, BDA-410 and E64) which showed protection in neurodegenerative diseases were systemically applied in the neonatal rats [29–31]. After injection for two days, cerebellum cytosolic calpain activity was significantly reduced in calpeptin- (55.6 ± 5.1%), SNJ1945- (66.6 ± 4.1%) and BDA-410- (53.7 ± 7.1%) treated rats (p<0.05), but not in E64-(102 ± 7.4%) treated rats (p>0.05) (One-way ANOVA, F (4, 18) = 10.41) (Figure 1B). Spectrin is one of the substrates of calpain, which was utilized to indicate calpain activity. As shown in Figure 1C, the level of spectrin breakdown products (SBPs) were significantly reduced in calpeptin-treated rats (*vs* control, 0.66 ± 0.06-fold) (Paired *t* test, p<0.05) (t = 6.67, df = 10.0). These data implicated that calpain activity in cerebellar tissue was inhibited by calpain inhibitors. In the subsequent experiments, calpeptin was selected to investigate the effects of calpain inhibition on adult behaviors and related mechanisms.

### Postnatal application of calpeptin attenuates suprachiasmatic nucleus circadian oscillatory protein (SCOP)-phosphorylated protein kinase B (p-AKT) pathway

**Figure 2:**
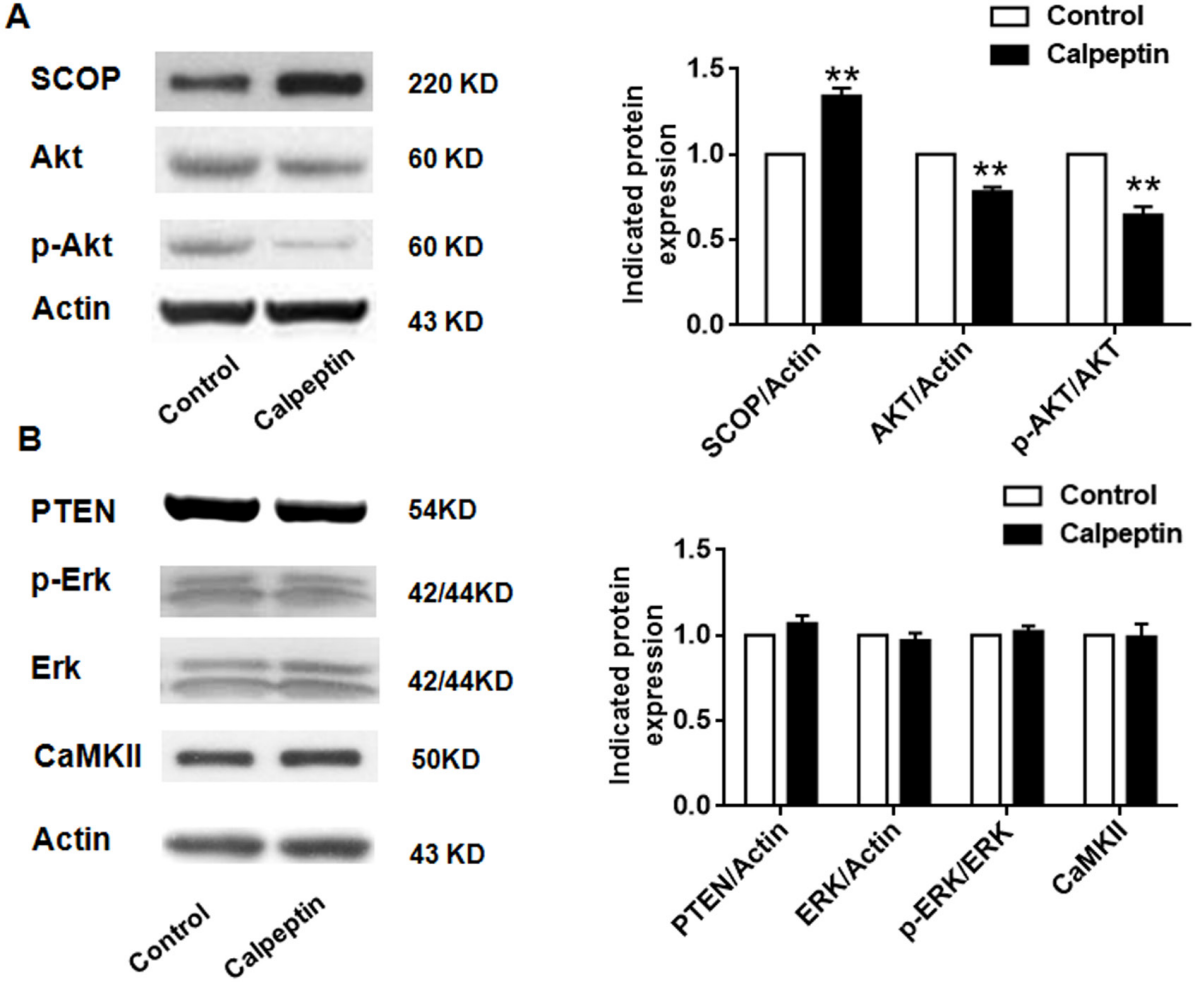
Postnatal application of calpeptin attenuates suprachiasmatic nucleus circadian oscillatory protein (SCOP)-phosphorylated protein kinase B (p-AKT) pathway **(A)** Expression of SCOP, p-Akt and Akt. Left panel: representative blots; Right panel: quantification data. **(B)** Expression of CaMKII, PTEN, ERK and p-ERK in cerebellum. Left panel: representative blots; Right panel: quantification data. Results represented means ± SEM (n = 5). ^**^p < 0.01 compared with control group (Paired *t* test).

Calpain functions mainly through degrading its substrates [32]. SCOP and phosphatase and tensin homolog (PTEN) were supposed as the classic substrates of calpains [26]. As shown in Figure 2A, calpeptin injection significantly promoted SCOP level compared with control (*vs* control, 1.36 ± 0.08-fold) (Paired *t* test, p<0.01) (t = 7.71, df = 8.0). However, calpeptin injection did not affect PTEN expression (*vs* control, 0.97 ± 0.03-fold, Paired *t* test, p>0.05) (Figure 2A). SCOP is a negative regulator of AKT and extracellular signal–regulated kinase (ERK) phosphorylation [22]. As shown in Figure 2A, 2B, calpeptin injection significantly decreased AKT phosphorylation (*vs* control, 0.66 ± 0.09-fold, Paired *t* test, p<0.05) (t = 15.71, df = 8.0), but did not affect ERK phosphorylation (Paired t test, p>0.05). Moreover, calpeptin injection decreased total AKT level (vs control, 0.79 ± 0.05-fold, Paired *t* test, p<0.05) (t = 13.51, df = 8.0), but did not affect total ERK and calmodulin-dependent Protein Kinase II (CaMKII) (Paired *t* test, p>0.05). These data might implicate that postnatal application of calpeptin specifically attenuated SCOP-AKT signaling pathway.

### Postnatal application of calpeptin promotes mammalian target of rapamycin (mTor) phosphorylation

**Figure 3:**
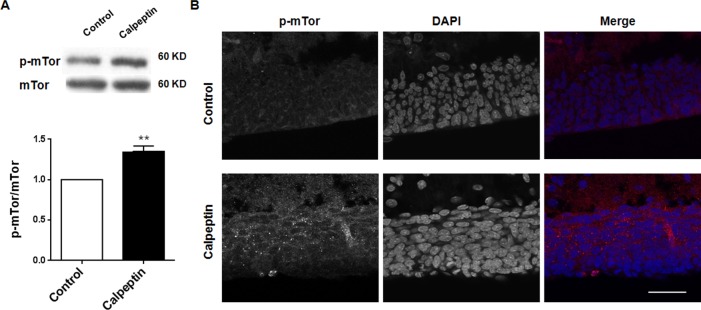
Postnatal application of calpeptin promotes mammalian target of rapamycin (mTor) phosphorylation **(A)** Upper panel shows representative blots of p-mTor in cerebellum. Lower panel shows summary data of p-mTor expression after application of calpain inhibitor. Results represent means ± SEM, n = 5. ^**^p < 0.01 compared with control group (Paired *t* test). **(B)** Representative images of p-mTor expression in cerebellum after injection of calpain inhibitor. Scale bar: 50 μm.

mTor pathway was supposed as a central link of signaling pathways involved in the cerebellar dysfunction [33]. We also detected mTor phosphorylation after calpeptin administration. As shown in Figure 3A, 3B, calpeptin injection significantly increased p-mTor level (*vs* control, 1.35 ± 0.07-fold, Paired *t* test, p<0.01) (t = 14.45, df = 8.0).

### Postnatal application of calpeptin promotes apoptosis of granular cells

**Figure 4:**
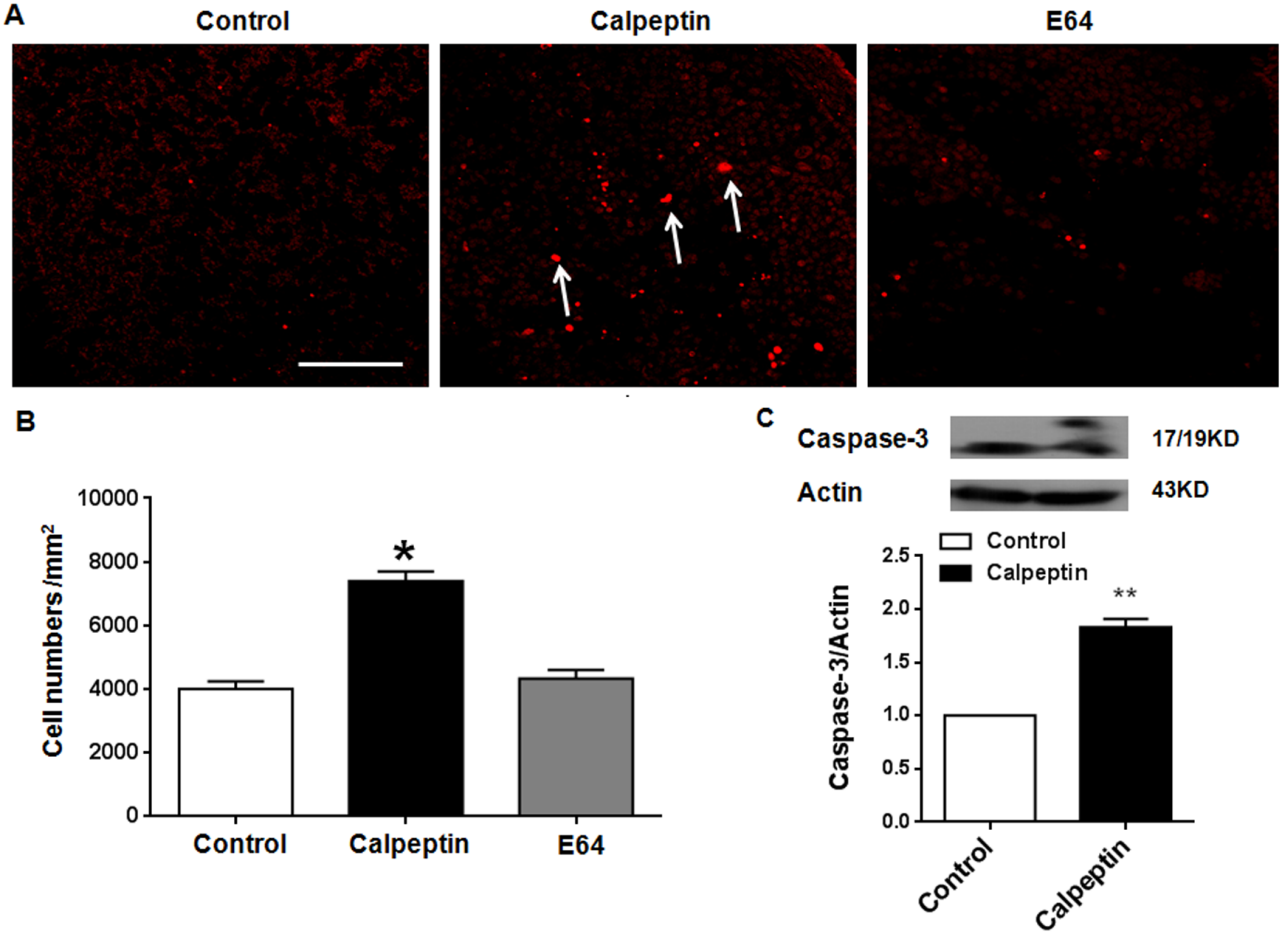
Postnatal application of calpeptin promotes apoptosis **(A)** TUNEL staining in cerebellar sections. Scale bar: 200 μm. **(B)** Quantification data of TUNEL positive cells. Results represent means ± SEM (n = 4). ^*^p < 0.05 *vs* control group. One-way ANOVA followed by Bonferroni test. **(C)** Upper panel shows representative blots for caspase-3. Lower panel shows summary data of caspase-3 expression after application of calpain inhibitor. Results represent means ± SEM (n=4-5). ^**^p < 0.01 compared with control group (Paired *t* test).

CAPN1 deletion caused apoptosis of cerebellar neurons [26]. In our study, we also detected apoptosis after calpeptin application. As shown in Figure 4A, 4B, calpeptin application for a period of 7 days significantly increased the apoptosis of granular cells in cerebellum (F (2, 15) = 8.98) (*vs* control, p<0.01). By contrast, similar application of E64 did not cause significant difference in apoptosis compared with saline (p>0.05). Caspase-3 is supposed as the apoptosis executor [34]. We detected cleaved caspase-3 level. As shown in Figure 4C, calpeptin injection remarkably increased the 19 kD- caspase-3 level, while did not affect 17 kD-caspase-3. We quantified both bands and results showed that calpeptin injection significantly promoted cleaved caspase-3 level (*vs* control, 1.83 ± 0.08-fold, Paired *t* test, p<0.01) (t = 17.31, df = 8.0).

### Postnatal application of calpeptin decreases the numbers of granular cells

**Figure 5:**
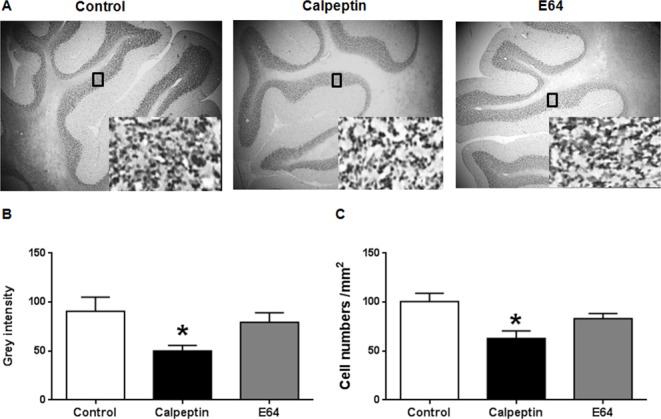
Postnatal application of calpeptin reduces the numbers of cerebellar granular cells **(A)** Representative images of Nissl staining. Insert are the images in high magnification. **(B)** Grey intensity in granular cell layer. **(C)** Cell numbers counted from high magnification images. Results represent means ± SEM (n = 4). ^*^p < 0.05 vs control group. One-way ANOVA followed by Bonferroni test.

Apoptosis was obviously increased after calpeptin application. We examined the changes of cell numbers in adult animals. As shown in Figure 5, calpeptin significantly decreased cell numbers in granular cell layer (F (2, 15) = 10.8) (*vs* control, p<0.05). By contrast, E64 did not affect the cell numbers compared with saline (p>0.05).

### Postnatal application of calpeptin alters the motor function

**Figure 6:**
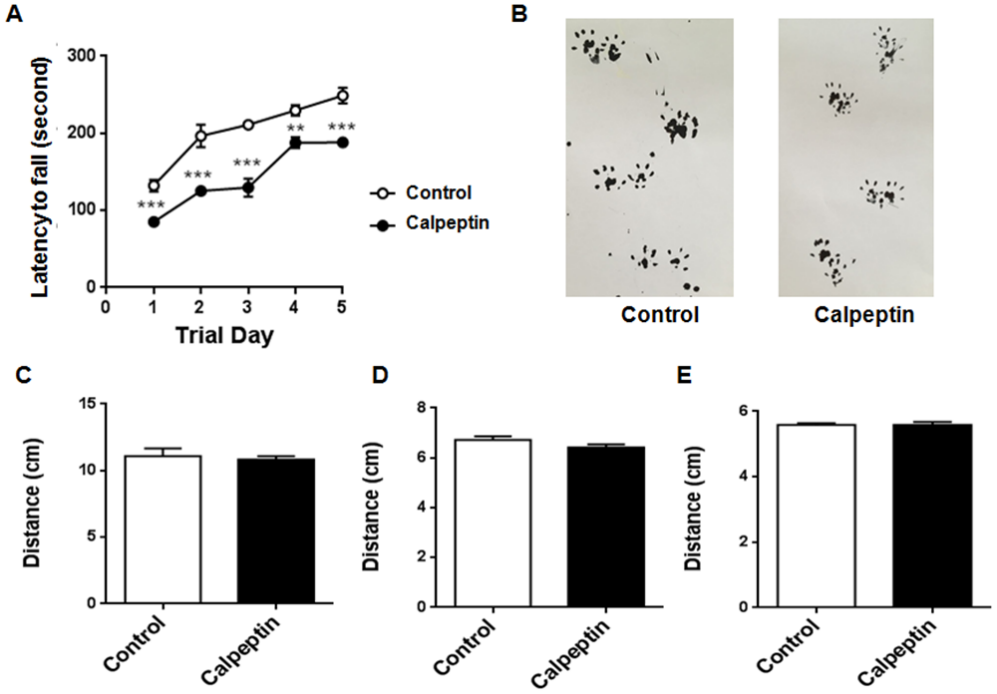
Postnatal application of calpeptin alters the motor function **(A)** Rotarod test of motor function. The latency to fall from an accelerating rotarod (from 4 to 40 rpm) was measured in four trials per day for 5 days. Results from the same day were averaged. Each value represents mean ± SEM (n = 8-13). ^**^p < 0.01, ^***^p < 0.001 compared with control group (Unpaired *t* test). **(B)** Representative footprints in control and calpeptin treated rats. **(C-E)** Stride, stance and sway lengths of control and modeled rats. Results were expressed as means ± SEM. n = 5-9.

Finally, we detected the behavioral changes in 4-month old animals. Rotarod test and gait experiments were applied to examine the cerebellum-related functions. As shown in Figure 6, motor function was apparently impaired in calpeptin-treated animals, while gait characters were not affected by calpeptin injection.

## DISCUSSION

In this study, we reported that postnatal application of calpain inhibitors were detrimental to cerebellar development. Different from other studies [16, 17], we demonstrated the protective action of calpain. The elimination of calpain in neonatal animals elicits abnormal development of cerebellum. Our findings support the previous reports that calpain could be neuroprotective in neurodevelopment [25, 26].

Calcium-related signaling pathways were required for neurodevelopment [35]. Moreover, there were evidences revealing that the activity calcium channel in neonatal animals is higher than that of adult animals [36]. In our study, we firstly distinguished the cytosolic calpain activities in different brain regions, including cortex, hippocampus and cerebellum. Consistent with previous publication [37], cerebellar calpain activity was the highest in the three detected brain regions. Interestingly, prenatal and postnatal period has the highest activity of calpain in whole life cycle [38], which implicates that calpain is important for early neurodevelopment.

Calcium overloading elicits calpain overactivation, which is proposed as an important pathological reason for neurodegeneration [39, 40]. In this present study, our data demonstrated that calpain inhibitors, including calpeptin, SNJ1945 and BDA-410 significantly reduced calpain activity in the postnatal rats. E64 is a broad spectrum cysteine proteinase and calpain inhibitor. However, in this present study, we did not find that E64 decreased cytosolic calpain activity in cerebellar tissue. The possible reason might be related to the chemical structure of that compound. The low permeability through blood brain barrier or the dose we used might be insufficient to inhibit cerebellar calpain activity. The special structural or chemical characteristic might also exclude the side effect of calpain inhibition on nervous system during the application of E64. In combination with previous publications [16, 17], we conclude that calpain inhibitors have the ability to eliminate the pathological activation of calpain, as well as physiological activation of calpain during neurodevelopment. The inhibition of physiological activation of calpain would contribute to neurodevelopment disorders.

CAPN1 deletion has been reported to influence the neurodevelopment in cerebellum [26]. In our study, we revealed that postnatal calpain was also critical for the cerebellar development. Although the specificity of the inhibitor cannot be neglected, calpeptin has been widely applied to investigate the calpain function [41], especially in central nervous system [42, 43]. Spectrin is one of the specific substrates of calpain [44], which are available to indicate calpain activity [45]. In our study, we evidenced that calpeptin injection decreased the spectrin breakdown, although spectrin is the skeleton protein which is required for sustaining cell morphology. The inhibition of spectrin breakdown might not be beneficial for cell proliferation, axon formation and reconstruction of dendritic spines. Besides the decrease of spectrin breakdown, calpeptin injection also promoted SCOP accumulation. Although SCOP is the negative regulator of AKT and ERK [22], p-AKT, but not p-ERK was decreased after calpeptin application. PTEN was also supposed as a calpain substrate [46]. However, in our study, we did not find obvious change of PTEN level after calpeptin injection. These data further implicate that different physiological action requires distinct calpain-mediated substrate degradation. As an example, we previously reported that 1-methyl-4-phenylpyridinium (MPP^+^) impaired hippocampal long-term depression via calpain-mediated degradation of protein tyrosine phosphatase 1B [45]. Moreover, PTEN was proposed as the substrate specifically cleaved by calpain-2 [46]. Although we did not distinguish the exact subtype of calpain in postnatal development, previous report to some extent implicated that calpain-1-mediated SCOP degradation is also responsible for postnatal cerebellar development [26].

mTor pathway serves a central link for several signaling pathways [47–49]. Moreover, mTor pathway was important for cerebellar development [50]. In our study, we found that calpeptin injection promoted mTor phosphorylation. However, one question is still left open. As AKT is an important upstream regulator of mTor [33], the decrease of p-AKT should also decrease p-mTor level. However, in this present study, we found the opposite result that calpeptin injection induced AKT inactivation, but mTor activation. It is difficult to explain that discrepancy based on the established signaling pathway [33]. However, there was one report implicating that calpain was important for autophagy during development [51]. Calpain activation decreased the autophagy activity. As mTor pathway is required for autophagy, the inhibition of calpain might promote p-mTor-mediated autophagy [52]. These data implicate other unknown regulatory mechanisms for m-Tor activation after calpain inhibition during cerebellar development.

In this study, we applied calpeptin for 7 days during the postnatal critical period. We found that the 4-month old rats in calpeptin-treated group showed motor deficit. Inconsistent with previous finding [26], we did not observe the deficit in gait experiments. These differences might be caused by the different animal strains used. Apoptosis, especially in granular cells was important for cerebellar development. In our study, terminal deoxynucleotidyl transferase (TdT)-mediated dUTP nick end labeling (TUNEL) assay and active caspase-3 further confirmed that both CAPN1 deletion and postnatal calpain inhibition could promote granular cell apoptosis.

Although CAPN1 deletion was reported to affect prenatal neurodevelopment, the postnatal effects of calpain on neurodevelopment were unknown. Our study utilized pharmacological approaches and verified the effects of calpain inhibitors on neurobehavioral activities and mechanisms involved. Different from previous publication [26], our study has important implications. Calpain activity is required for postnatal neurodevelopment, which implicates that direct blockage of calpain activity in young animals will pay price for the neurodevelopment. Therefore, the therapeutic target for neurodegenerative diseases should be the specific substrate of calpain rather than arbitrary blockage of calpain [53]. Although the effect of postnatal application of calpain inhibitors on other behavioral phenotypes is still under investigation, the detrimental activity of postnatal calpain application should take more attentions. In addition, besides SCOP-AKT pathway, mTor pathway might also be involved in calpeptin-mediated apoptosis or behavioral deficit.

In our study, we demonstrated that calpain inhibitors attenuated the calpain activity, which was detrimental to cerebellar development. The potential mechanisms might be related to apoptosis of granular cells via SCOP-AKT and mTor pathways.

## MATERIALS AND METHODS

### Animals and treatments

Male and female adult Sprague Dawley rats were obtained from the Animal Center of Anhui Medical University (Hefei, China) and housed in a temperature-controlled room with a standard 12-h light/12-h dark cycle and *ad libitum* access to food and water. This study was carried out in accordance with the recommendations of Anhui University of Traditional Chinese Medicine. The protocol was approved by the Ethics Committee of Anhui University of Traditional Chinese Medicine. All efforts were made to minimize the number of animals used and their suffering. Except for the brief intervals of separation required for daily injection, the pups were kept with their dams throughout the experiment. Control and experimental pups were obtained randomly from the same litters. Two rats were bred in one plastic cage (40 × 20 × 18 cm).

The birth day was designated as P0, pups received 2 mg/kg calpeptin (sc-202516), SNJ1945 (1 mg/kg), BDA-410 (1 mg/kg) or E64 (5 mg/kg) intraperitoneally (*i.p.*) on P7 and P8. The cerebellar tissue was isolated for biochemical experiments two h after injection of calpain inhibitors. An equivalent volume of saline was used as control. After a period of 7-day injection of calpeptin (one injection per day), the cerebellar tissue was isolated and fixed in 4% paraformaldehyde for apoptosis detection. Four months after application of calpain inhibitor (a period of 7-day injection), behavioral experiments and Nissl staining of cerebellar tissue were carried out. TUNEL assay, p-mTor and Nissl staining were conducted in 9 sections at 1.9, 1.4, 0.9, 0.4, −0.1, −0.6, −1.1, −1.6 and −2.1 Bregma from each cerebrum.

### Calpain activity assay

Cerebellum, cortex and hippocampus were obtained from P8 rats after decapitation. Calpain activity in different brain area was detected. In other part of experiment, cerebellar tissue was collected two h after two-day application of calpain inhibitors. Cytosolic calpain activity was detected following the instruction of kit (K240-100, Biovision, USA). Briefly, reaction mix was added and incubated with the homogenation at 37 °C for 1 h after homogenation. The fluorometric assay is based on the detection of cleavage of calpain substrate Ac-LLY-4-trifluoromethylcoumarin (AFC) (λmax = 400 nm). Upon cleavage of the substrate by calpain, free AFC emits a yellow-green fluorescence (λmax = 505 nm), which could be quantified using a Fluorescence Plate Reader (Thermo Fisher Scientific, USA). RFU/10 mg tissue was calculated.

### Immunochemical staining

After fixation in 4% paraformaldehyde for 1 h, cerebellar tissue was cryoprotected in 30% sucrose for 1 h at 4°C and sectioned on a freezing microtome at 20 μm. Sections were blocked in 0.1 M phosphate buffer saline (PBS) containing 10% goat serum and 0.4% Triton X-100, and then incubated with primary antibody (mouse anti-p-mTor, 1:1000, Santa Cruz Biotechnology) in 0.1 M PBS containing 5% goat serum and 0.4% Triton X-100 overnight at 4°C. Sections were washed three times (15 min each) in PBS and incubated with Alexa Fluor 593 goat anti-mouse IgG (Life Technologies) for 2 h at room temperature. The images were taken by FV1000 Olympus Confocal Laser Scanning Microscope (Olympus, Japan).

### Western blotting

Cerebellar homogenates from five animals were obtained from each group and lysed. The Bicinchoninic Acid Kit (Beyotime Institute of Biotechnology, Shanghai, China) was used to detect protein concentration. Protein samples were separated on sodium dodecyl sulfate polyacrylamide gel electrophoresis (SDS-PAGE) (12% gel) for 50 min at 120 V and transferred onto nitrocellulones membrane for 35 min at 300 mA. The membranes were blocked with Tris-buffered saline (TBS), containing 0.1 % Tween 20 (TBST) and 5 % fat-free milk for 2 h at room temperature and then incubated (overnight, at 4°C) with the rabbit antibodies against Spectrin (1:1000, Santa Cruz Biotechnology), SCOP (1:1000, Santa Cruz Biotechnology), p-Akt (1:1000, Cell Signaling Technology), Akt (1:1000, Cell Signaling Technology), CaMKII (1:1000, Cell Signaling Technology), PTEN (1:1000, Cell Signaling Technology), p-ERK (1:1000, Cell Signaling Technology), ERK (1:1000, Cell Signaling Technology), active Caspase-3 (1:1000, Cell Signaling Technology), p-mTor (1:1000, Santa Cruz Biotechnology) and Actin (1:500, Zsbio, Beijing, China) followed by a 2-h incubation with a peroxidase-conjugated affinipure goat anti-rabbit immunoglobulin G (1:5000, Zsbio, Beijing, China) at room temperature. The intensity of the band was quantified by densitometry using ImageJ software (NIH, Bethesda, MD, USA). Quantification of total protein was determined relatively to Actin, whereas phospho-protein was determined relatively to total protein for the same experiments. Ratios of total protein/Actin or phosphor-protein/total protein in experimental groups were obtained for each experiment and normalized to control to avoid the variation within group.

### TUNEL staining

TUNEL staining was performed in 30-μm cerebellar slices using the ApopTag *In Situ* Apoptosis Detection Kit (C1089, Beyotime Institute of Biotechnology, Shanghai, China) following the manufacturer's instruction. After staining, the sections were imaged using FV1000 Olympus Confocal Laser Scanning Microscope (Olympus, Japan). For cerebellar analysis, the outline of the cerebellum in each image was drawn using “free hand selections” in ImageJ. TUNEL-positive nuclei in cerebellar area were counted using “particle analysis” in ImageJ software.

### Rotarod test

Rotarod test was conducted following the protocol as previously described [54] using a rotarod apparatus (IITC, CA, USA). The total test period lasted 6 days (1 training day followed by 5 trial days). The rotor was set at a constant speed of 4 rpm, and animals were placed on the rod for 30 s on day 1. If the animals fell off the rod prior to the end of the 30 s, they were placed back. Trial days consisted of three trials (5 min in each trial). In the experiment, the rod was set to ramp up from 4 to 40 rpm over the time course of 5 min. When the animal fell off the rod or at the end of 5 min, the latency to fall was recorded. Each animal had a 15-min interval rest between trials.

### Gait analysis

Rats were trained for one day to walk through the tunnel and then tested for two trials. Four steps from the middle portion of each run were analyzed for hind-stride length and hind-base width (distance between the right and left hind-limb strides, sway distance).

### Nissl staining

Cerebellar tissues were fixed in 4% paraformaldehyde for 1 h. After that, the tissues were cryoprotected in 30% sucrose for 1 h at 4°C and sectioned on a freezing microtome at 20 μm. Sections were blocked in PBS and stained in cressyl violet for 3-5 minutes, then rinsed in distilled water and soaked in 95% ethyl alcohol for 15 seconds. The images were taken under light microscope. Four images were taken from each slice. The grey value in the images represented the cell density of granular cells. The images were saved in black/white format and the grey values were analyzed using ImageJ software. Alternatively, the granular cells were counted in the high magnification images. At least two fields in each image were counted and averaged.

### Statistical analyses

Data were presented as means ± SEM and analyzed by GraphPad Prism 6.0. Two-tailed student t-test or one-way ANOVA was applied to determine statistical significance. A value of *p*<0.05 was considered to be significant.

## Abbreviations

SBPs: spectrin breakdown products
RFU: relative fluorescence unit
p-Akt: phosphorylated protein kinase B
SCOP: suprachiasmatic nucleus circadian oscillatory protein
